# 

**Published:** 1891-12-26

**Authors:** 


**Notices of Births, Deaths, and Marriages**, are inserted in this column at a charge of 2s. 6d. for an announcement not exceeding four lines.

### NOTICE TO CORRESPONDENTS.

All MS., Letters, Books for Review, and other matters intended for the Editor should be addressed The Editor, The Lodge, Porchester Square, London, W.

The Editor cannot undertake to return rejected M.S., even when accompanied by stamped directed envelope.

**Notice to Advertisers and Subscribers.**

All Advertisements, Orders for Copies of The Hospital, and Business Communications should be addressed to The Publisher, *not to the Editor*, The Hospital, 140, Strand, W.C.

The Cost of Subscription, post free, is as follows:—Per week, 1½d.; per quarter, 1s. 8d.; per half-year, 3s. 3d.; per annum, 6s. 6d. Foreign:—Per annum, 8s. 8d.; payable, in all cases, in advance.

**"Burdett's Hospital Annual," 1891—1892.**

Third year of publication. 600 pp. Boards, gilt lettering. A most complete guide to British and Colonial Hospitals, Dispensaries, Nursing and Convalescent Institutions, and Asylums. Price 3s. 6d. Published by The Hospital (Limited), 140, Strand, W.C.

**NOTICE.—The Index for Vol. X. (ending September, 1891), is on ale at the Office of this Journal, price 2½d., post free.**

### SCRAPS AND GLEANINGS.

THE Duchess of Albany opened a bazaar at the Village Hall, Weybridge, in support of the Clerical Aid Society.

HER Majesty the Queen has been graciously pleased to accept a copy of Gertrude and Ethel Armitage Southam's book, entitled "Hors de Combat; or, Three Weeks in a Hospital."

THIRTY thousand cases of influenza are stated to have occurred in Lemburg since October, and other Galician towns are equally affected. The malady is making steady progress in Vienna.

THE Glynde Convalescent Home was opened on the 26th ult. by the Hon. Mrs. T. Brand. It is intended solely for young children, and is built upon a site near Eastbourne, given by Lord Hampden.

THE death is announced of Julia Cronin, who died at Ballymount, near Killarney, at the extraordinary age of 115 years. This is the second centenarian who has died at Killarney within the past month.

J. B. WALKER, Lieut.-Colonel, Hon. Secretary Church of England Soldiers' Institute, makes an appeal for Christmas-cards for the soldiers in the military hospitals at Aldershot. Cards that have been written on are of no use.

A BAZAAR in aid of St. Faith's House of Mercy, Lostwithiel, was held during the last week of November. Sister Anna, who has managed the House of Mercy since its opening is held in high esteem by the inhabitants of Lostwithiel.

THE outbreak of diphtheria at Bishopstoke, Hampshire, has spread to the surrounding villages. The Board School at Fairoak has been closed, and utilised as a hospital. There are at present about twenty patients there. Three children have already died.

THE passenger railway employés at Newcastle and Gateshead stations, numbering 352 men, held a meeting on Sunday, at which it was resolved that a half-day's pay per man each year be devoted to the medical charities. This will be taken in January, and will realise £30 or £40.

THE "Hibernia" is a three-decker, and being a wooden ship, some 95 years old, her timbers are in a fit state to harbour microbes and fever germs. It is strange that the Admiralty do not establish naval barracks here. There are at present living on board the "Hibernia" 740 men.

AT a meeting of the Board of Management of the Salford Royal Hospital, held in the Board-room of the Institution on Wednesday, the 9th inst., Mr. G. A. Southam in the chair, the Chairman announced the receipt of £1,000 from the Residuary Legatees of the late Mrs. Sykes, of Blackpool.

THERE are very many sick in the Naval Hospital at Malta. An extra surgeon has been appointed, and also an extra sister, within the past few days. Some people say the sickness is due to the overcrowded state of the receiving ship "Hibernia," flag-ship of Rear-Admiral Buller, C.B.

SUNDERLAND'S CHARITY proceeds from a bequest by Samuel Sunderland in 1671 of 22 acres of land, the rent from which was to be distributed amongst the poor of Bingley. The value of the bequest has increased to the extent which enables the trustees to endow the Cottage Hospital.

AT a meeting of the Shipwrecked Mariners' Society, held at 28, Great George Street, the reports presented showed that altogether the charity had helped, throughout the year, 5,409 cases of direct shipwreck distress, belonging to some 686 vessels, the total relief disbursements amounting to £17,645.

SIR W. HOULDSWORTH, speaking at the annual meeting of the Hospital Sunday and Saturday Funds at Manchester, remarked, and we think truly, that "There is reason to believe there are still thousands of persons not ill-disposed to charities who are never reached by the ordinary canvassers, nor by collections in churches or chapels."

THE National Society for the Prevention of Cruelty to Children have, during the last recorded month, investigated 713 complaints of ill-treatment and neglect affecting the welfare of 1,735 children. In 426 cases warnings were given; in 87 the offences were so gross as to necessitate prosecutions (percentage of convictions 94·25), the rest being dealt with in other ways.

A CONFERENCE of Guardians and members of the Stockport Town Council took place at the Stockport Union Workhouse yesterday to consider a proposal to erect a hospital for the reception of pauper infectious patients in Stockport Union. After considerable discussion it was decided to accept the offer of the Corporation to provide accommodation for the purpose, the Guardians to pay 7½d. per cent. of the cost.

WELLINGTON COLLEGE was closed none too soon. Colonel Moysey is mourning the loss of a son within five days of leaving the school, and this, it is said, is not an isolated instance. The advice of Dr. Thorne Thorne, of the Local Government Board, may be commended to all schoolmasters. In his recently-published work Dr. Thorne says diphtheria is a disease to which "school influence" contributes extensively.

INFLUENZA is at last officially admitted to be in Paris. Professor Brouardel stated at the Academy of Medicine that the death rate has risen 100 within the last week. This increased mortality is due to the epidemic on its secondary effects. Influenza is still rife, and not in a mild form, in the Central and South-Western Departments. Doctors Sée and Beaumetz warn people to avoid fatigue, to be careful about their food, and to keep themselves warm.

A CONCERT in aid of the Irish evicted tenants was held at the Stratford Town Hall. Mr. William Ludwig and other popular Irish singers rendered a choice selection of Irish national music. The concert is the second of a series which is being held in London under the auspices of the Irish National League of Great Britain. The former concert, which was held in Kensington, resulted in a substantial sum being forwarded to the Secretaries of the Evicted Tenants' Fund.

THE Earl of Strafford presided at the half-yearly meeting of the East London Hospital for Children and Dispensary for Women. Upwards of 22,000 old and new patients have been treated. In the wards of the hospital 551 children had been nursed. The total amount received had been £6,789, compared with £5,200 in 1890. The building of the new out-patient rooms was begun in June, the cost being £7,500, towards which £5,600 had been subscribed. Donations are asked for in order to open the building free of debt.

### The Student of Medicine.

[All communications for this Supplement should be addressed to The Editor of The Hospital, at 140, Strand, with the words, "Students' Column," written in the left hand top corner of the envelope.]

### APPOINTMENTS.

At the Clayton Hospital, Wakefield, John W. Bone, M.B. C.M. Edin., has been appointed junior house surgeon. At the Brighton and Sussex Throat and Ear Hospital, Herbert Connop, B.A. Camb., L.R.C.S., L.R.C.P. Edin., has been appointed house surgeon. At the North Staffordshire Infirmary and Eye Hospital, J. Frank Crombie, M.B., C.M. Edin., has been appointed assistant house surgeon. At the Queen's College, Birmingham, F. H. Goffe, L.D.S.,R.L.S., has been appointed a demonstrator of medical dentistry. At the Great Northern Central Hospital, P. Traer Harris, M.R.C.S., L.D.S., has been appointed dental surgeon. At the Paddington Infirmary, Robert D. Hotchkis, M.A., M.B., B.S. Durham, has been appointed resident clinical assistant. At the Royal Victoria Hospital, Bournemouth, F. Winson Ramsay, M.D., B.S. Durham, L.S.A. Lond., has been appointed honorary medical officer. At the Royal Hospital for Diseases of the Chest, City Road, Gustave Shorehem, M.A., M.B., B.ch. Oxon., M.R.C.P., has been appointed assistant physician. At the London Hospital, Frederick John Smith, B.A., M.B., M.R.C.P., F.R.C.S., has been appointed assistant physician.

### VACANCIES.

The following vacancies are announced this week, the amount of salary in each case being given after the name of the institution:

### Resident Medical Officers.

General Hospital, Birmingham, House Surgeon—Board, &c.

Hospital for Consumption, Brompton, House Physician—Board, &c.

Liverpool Northern Hospital, Assistant House Surgeon—Salary £70, with board, &c.

North Staffordshire Infirmary and Eye Hospital, Harts Hill, Stoke-upon-Trent, House Physician—Salary £100, with board, &c.

Oldham Infirmary, Junior House Surgeon—Salary £50, with board, &c.

Sunderland Infirmary, House Surgeon—Salary £80, with board, &c.

Victoria Hospital for Sick Children, Chelsea, House Surgeon; also House Physician—Salaries £50, and board, &c.

York County Asylum, Junior House Surgeon—Salary £40.

### Non-Resident Medical Officers.

General Hospital, Birmingham, Honorary Surgeon.
General Infirmary at Gloucester and the Gloucester Eye Infirmary, Surgeon.
Owens College, Manchester, Professor of Botany.
University College, Dundee, Demonstrator of Anatomy—Salary £120.

### PASS LISTS.

### University of London.

The following candidates passed the M.B. Examination in October last:—

First Division.—W. L. Andriezen, University College; G. F. Blacker, University College; E. E. Blomfield, London Hospital; G. S. Buchanan, B.Sc., St. Bartholomew; H. A. Caley, St. Mary's; C. C. Chidell, University College; G. H. Cooke, Manchester Royal Infirmary and Owens College; T. P. Cowen, St. Thomas's; H. J. Curtis, University College; T. B. P. Davis, Guy's; D. Drew, University College; A. E. Giles, B.Sc., Owens College and Manchester Royal Infirmary; J. C. M. Given, Royal Infirmary School of Medicine, Liverpool; A. Griffith, University College; H. Hodgson, Guy's; B. W. Hogarth, Guy's; H. Horrocks, B.Sc., Owens College and Manchester Royal Infirmary; R. E. S. Krohn, University College; A. Manknell, Yorkshire College; Elizabeth M. Pace, London School of Medicine and Royal Free Hospital; J. E. Paul, University College; A. T. Rake, Guy's; H. M. Richards, University College; A. G. Rider, University College; G. A. Simmons, St. Mary's; E. N. Smith, University College; C. R. Stevens, St. Bartholomew's; W. M. Stevens, University College; Mary D. Sturge, London School of Medicine for Women; W. F. Umney, St. Thomas's; E. E. Ware, St. Thomas's; A. S. Wohlmann, Guy's; Emily E. Wood, London School of Medicine and Royal Free Hospital; J. Young, Guy's.

Second Division.—C. F. M. Athorp, Yorkshire College; Vivian B. Bennett, University College, Liverpool; E. H. Biddlecombe, St. Bartholomew's; J. A. Codd, B.Sc., St. Thomas's; H. A. Eccles, St. Bartholomew's; W. W. Kennedy, St. Bartholomew's; M. Nicklin, Queen's College, and General Hospital, Birmingham; G. D. Parker, St. Bartholomew's, C. H. Preston, Owens College, and Manchester Royal Infirmary; E. L. Pritchard, King's College; C. E. Salter, Guy's; F. P. Sarjant, Guy's; A. E. Tebb, Guy's; A. Thomas, Guy's; Ethel M. N. Williams, London School of Medicine for Women; J. G. Wilson, London Hospital.

